# Early onset X‐linked female limited high myopia in three multigenerational families caused by novel mutations in the *ARR3* gene

**DOI:** 10.1002/humu.24327

**Published:** 2022-01-19

**Authors:** Ralph van Mazijk, Annechien E.G. Haarman, Lies H. Hoefsloot, Jan R. Polling, Marianne van Tienhoven, Caroline C.W. Klaver, Virginie J.M. Verhoeven, Sjoukje E. Loudon, Alberta A.H.J. Thiadens, Anneke J.A. Kievit

**Affiliations:** ^1^ Department of Clinical Genetics Erasmus Medical Centre Rotterdam The Netherlands; ^2^ Department of Ophthalmology Erasmus Medical Centre Rotterdam The Netherlands; ^3^ Department of Epidemiology Erasmus Medical Centre Rotterdam The Netherlands; ^4^ Department of Ophthalmology Radboud University Medical Center Nijmegen The Netherlands; ^5^ Institute of Molecular and Clinical Ophthalmology University of Basel Basel Switzerland

**Keywords:** atropine, genetics, Mendelian, myopia, refractive error, syndromic myopia

## Abstract

This study describes the clinical spectrum and genetic background of high myopia caused by mutations in the *ARR3* gene. We performed an observational case series of three multigenerational families with high myopia (SER≤−6D), from the departments of Clinical Genetics and Ophthalmology of a tertiary Dutch hospital. Whole‐exome sequencing (WES) with a vision‐related gene panel was performed, followed by a full open exome sequencing. We identified three Caucasian families with high myopia caused by three different pathogenic variants in the *ARR3* gene (c.214C>T, p.Arg72*; c.767+1G>A; p.?; c.848delG, p.(Gly283fs)). Myopia was characterized by a high severity (<−8D), an early onset (<6 years), progressive nature, and a moderate to bad atropine treatment response. Remarkably, a female limited inheritance pattern was present in all three families accordant with previous reports. The frequency of a pathogenic variant in the *ARR3* gene in our diagnostic WES cohort was 5%. To conclude, we identified three families with early onset, therapy‐resistant, high myopia with a female‐limited inheritance pattern, caused by a mutation in the *ARR3* gene. The singular mode of inheritance might be explained by metabolic interference due to X‐inactivation. Identification of this type of high myopia will improve prompt myopia treatment, monitoring, and genetic counseling.

## Graphical abstract

This study describes the clinical spectrum and genetic background of high myopia caused by mutations in the ARR3 gene. A mutation in this gene causes high myopia with a female‐limited inheritance pattern, probably caused by metabolic interference due to X‐inactivation.

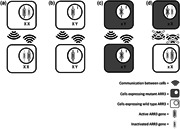

## 1 INTRODUCTION

Myopia is a refractive error in which light rays entering the eye are not focused correctly on the retina leading to blurred vision which can be treated with glasses or contact lenses (Flitcroft et al., 2019). Later in life, however, myopia and especially high myopia, defined as a refractive error of more than −6 diopters (D), can lead to irreversible visual impairment or blindness due to its complications including glaucoma, retinal detachment, and most importantly myopic macular degeneration (Fricke et al., 2018; Verhoeven et al., 2015). The incidence and prevalence of (high) myopia has increased dramatically around the globe (Holden et al., 2016). Given the consequences of this common ocular condition for the patient but also considering the (financial) disease burden on our health‐care system, there is a need for proper understanding of its etiology and need for therapy.

Based on its severity degree, occurrence and genetic background, common myopia is distinguished from Mendelian myopia. The former is often less severe and caused by a complex interplay between genetic factors and environmental influences such as near work and outdoor activity. These genetic factors, of which more than 500 have been identified up till now, are common and have small effect sizes but the sum of these factors and the environmental risk factors increase the risk of becoming myopic or even highly myopic (Tedja et al., 2018). The latter is frequently more severe, with earlier age of onset and caused by rare genetic variants with an autosomal dominant, autosomal recessive or X‐linked inheritance pattern and even X‐linked female limited. Among this type are isolated, but also syndromic forms of myopia accompanied by ocular or systemic features as well as retinal dystrophies co‐occurring with myopia (Tedja et al., 2019).

To pinpoint a specific genetic cause of myopia an extensive work‐up is advised which includes asking for certain symptoms (e.g., night blindness and joint laxity), drawing a family pedigree, and conducting a physical and ocular examination with attention to special features which could highlight a certain underlying genetic defect. With this study‐up, an ophthalmologist or clinical geneticist can identify a specific cause of myopia which may have implications for diagnosis, therapy and prognosis for the patient, but also for its relatives. In this report we describe three early onset high myopia Dutch families with a rare but unique and recognizable inheritance pattern caused by a pathogenic *ARR3* variant and provide new insights in addition to previous reports on this type of high myopia (Széll et al., 2021; Xiao et al., 2016).

## 2 METHODS

### 2.1 Editorial policies and ethical considerations

Informed consent was obtained from all patients and their parents if applicable.

### 2.2 Patient population

Patients were included at the outpatient clinics of the departments of Clinical Genetics and Ophthalmology at the Erasmus Medical Center in Rotterdam, the Netherlands. Data from medical charts were collected retrospectively from all patients and were compared with the findings described before in other families (Xiao et al., 2016). Genomic DNA was used for further molecular studies.

### 2.3 Genetic testing

Genomic DNA was extracted from lymphocytes of 5 ml samples of EDTA anticoagulated blood according to standard protocols. Whole‐exome sequencing (WES) was performed as described earlier (Oegema et al., 2017). In short, exome‐coding DNA was captured with the Agilent Sure Select Clinical Research Exome SureSelect kit (V4 (03‐2015), CRE V1 (03‐2015 to 03‐2017), CRE V2 (03‐2017 to 12‐2019) and V7 (12‐2019 to now)). Reads were aligned to Hg19 and variants were called using the GATK haplotype caller (v2.7‐2). Detected variants were annotated, filtered, and prioritized using Alissa Interpret (formerly Cartagenia Bench Lab NGS). Filter steps included a gene panel restricted to genes associated with vision disorders such as retinal dystrophies, connective tissues diseases, or corneal disorders (Whole Exome Sequencing Gene package Vision disorders, 2019) (details available on request), followed by a full open exome sequencing without this gene filter and slightly different settings. Identification of a possible causative variant was confirmed by Sanger sequencing. Segregation analysis was performed using targeted sequencing of the familial variant in relatives.

## 3 RESULTS

We identified three Caucasian families with high myopia caused by a pathogenic variant in the *ARR3* gene. Myopia occurred at an early age (<6 years) and was severe (<−6D). A detailed description of the families can be found below.

### 3.1 Clinical presentation and family history

**Figure 1:**
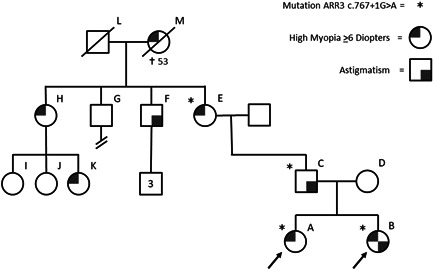
Pedigree of family I. Patient A, B, and their father have confirmed mutations in the ARR3 gene. Great‐uncle H has astigmatism, he has three sons, and none of them has confirmed high myopia. Great‐uncle G had no visual complaints and was childless, great‐aunt F is known with high myopia and has three daughters of which one is also affected by high myopia. Great‐grandmother M had also been affected with high myopia

In the first family (Family I) two little sisters of nonconsanguineous Caucasian parents were referred to the ophthalmologist because of early onset high myopia (Figure 1 and Table 1). Sister A (6 years) had a spherical equivalent of refraction (SER) in cycloplegia of −8.0/−9.5 D and an axial length (AL) of 26.75/26.09 mm. The younger sister B had a cycloplegic refraction of −8.0/−8.75 D and AL 26.09/26.08 mm at 4 years of age. Other ophthalmological screening, including inspection of the anterior segment and fundocopic examination showed no abnormalities. Myopia was highly progressive in both sisters, being more than 1D progression per year. Therefore, both sisters were treated with high‐dose atropine eye drops (0.5%), and the use of multifocal and photochromatic glasses. No systemic or local adverse events were reported so patient adherence remained high. Nevertheless, progression was evaluated after 1 year and remained high (annual increase −1.0/−0.75 D and 0.84/0.79 mm for A; −1.25/−2.0 D and 0.73/0.94 mm for B).

**Table 1:** Clinical findings in the three ARR3 families

Person (symbol pedigree)	Mutation *ARR3*	SER (Diopters)	Astigmatism	Axial length (mm)	Complications
Family I	NM_004312.2(*ARR3*) c.767+1G>A; p.?.				
A	+	−8/.0‐9.5		26.75/26.09	
B	+	−8.0/−8.75	+	26.09/26.08	
C	+	‐/‐	+		
E	+	−20/−20			Myopic macular degeneration, glaucoma, subcapsular cataract
F		‐/‐	+		
H		−19/−20			
K		<−6/<−6			
M		−13/−15			Posterior subcapsular cataract, subretinal neovascularization, ocular hypertension
Family II	NM_004312.2(*ARR3*) c.214C>T, p.(Arg72*)				
A	+	−18.75/−16.00		30.46/29.70	Myopic macular degeneration
C		−24/−24			
D	+	−12/−12			
G	+	−10/−10			
L		<−6/<−6			Retinal detachment
M		−23/−23			
N		<−6/<−6			
O		<−6/<−6			
Family III	NM_004312.2(*ARR3*) c.848delG, p.(Gly283fs)				
A	+	−15.75/−11.00		29.40/28.03	Ocular migraines, recurrent nasotemporal vision loss. Posterior staphyloma (right eye)
B		−2/−1			
C	+	−20/−20			
D		<−6/<−6			
E		−6/−6			
F		−1/−1			
G		−3/−3			
H		<−6/<−6			

Abbreviation: SER, spherical equivalent of refraction.

Family history revealed that in addition to the probands, several other female members (E, H, K, and M) suffered from early onset high myopia (Figure 1). Ophthalmological findings in the other female relatives showed early onset progressive myopia (mean SER of −18 D) and different myopic complications including cataract (E and M), myopic macular degeneration (E and M), and glaucoma (E) in affected females (Table 1). Several male relatives (C, F) had astigmatism and mild myopia, but none showed early onset high myopia. The pattern of affected females in these families and possibly mildly affected males in family I followed a mode of inheritance known as X‐linked female limited. However, at the time there was no known genetic cause of early onset high myopia following this inheritance pattern.

In family II, a 9‐year‐old girl of nonconsanguineous Caucasian parents was referred for early onset high myopia (SER −18.75/−16.00 D and AL 30.46/29.70 mm). She had visual correction with glasses since she was a baby. No abnormalities were found during anterior segment examination; fundoscopic examination showed myopic macular degeneration in both eyes. Since her myopia was progressive, she was treated with high‐dose atropine 0.5%. Due to insufficient response, this was increased to atropine 1% after 6 months.

**Figure 2:**
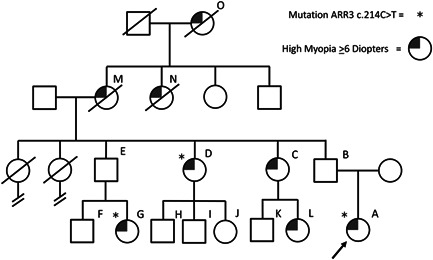
Pedigree of family II, index patient A has confirmed mutations in the *ARR3* gene. Aunts D and C have high myopia. Nieces G and L have high myopia; moreover, niece G suffered Retinal detachment at age 18. Deceased grandma M and her deceased mother O and her deceased sister N all were affected by high myopia. The familial pathogenic variant was segregated in aunt D and niece G

Family history also revealed a number of affected female family members (mean SER of −17 D) (Figure 2 and Table 1). A niece (K) had suffered from a rhegmatogenous retinal detachment. No male relatives were suffering from early onset high myopia nor astigmatism.

In the last family (III), an adult woman of nonconsanguineous Caucasian parents presented with recurrent sudden vision loss in the inferior nasal temporal quadrant. This vision loss occurred after waking up and was accompanied by headaches and the sensation of flickering lights. Her medical history included early onset high myopia (SER −15.75/−11.00 D and AL 29.40/28.03 mm), which was diagnosed at age 6 and a nystagmus which was recognized even at an earlier age. Fundoscopic examination did not reveal any abnormalities except for staphyloma (Figure S1). Fluorescein angiography showed no signs of vascular occlusion or hemorrhage. Goldmann visual field examination confirmed the anamnestic vision loss. Magnetic resonance imaging (MRI) of the orbits showed a stretched bulbus oculi with staphyloma posterior in the right eye. As the headaches were accompanied by the sensation of flickering lights, her vision loss was most likely caused by ocular migraines.

**Figure 3:**
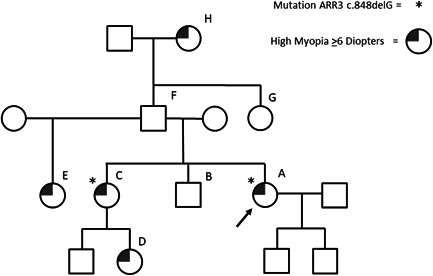
Pedigree of family III. Patient A has a confirmed mutation in the *ARR3* gene. Sister C has high myopia; her 1‐year‐old daughter (D) has progressing early onset high myopia. Half‐sister E also suffered from high myopia. Grandma H was also been afflicted with high myopia

Family history included several females whom were affected by early onset high myopia (mean SER of −15 D) (Figure 3 and Table 1). No male relatives were affected by astigmatism nor high myopia.

### 3.2 No clues for a syndromic cause of myopia

None of the index patients showed dysmorphic features, disproportion, joint laxity, deafness and/or learning disabilities, consistent with syndromic high myopia, like Marfan, Stickler, Knobloch, or Weill‐Marchesani syndrome. Variants in the associated genes were not found. Therefore, it was considered an isolated form of familial early onset high myopia in all cases.

### 3.3 Genetic diagnosis

In family I initially WES with a filter for vision disorders was performed on DNA from sister B and her parents, which did not demonstrate pathogenic variants explaining the clinical presentation. Genetic testing was expanded to a full open exome, which also did not reveal any known pathogenic variants. A PubMed search resulted in an article by Xiou et al., which describes a X‐linked female limited inheritance pattern caused by an *ARR3* mutation (Xiao et al., 2016). Based on this article it was decided to perform an additional analysis of the *ARR3* gene on the X‐chromosome because this gene was not in the panel for vision disorders and the paternal inherited mutation on the X chromosome had not been prioritized, due to the rare inheritance pattern.

Additional review of the X‐chromosome revealed a paternally inherited pathogenic splice site mutation variant in the *ARR3* gene NM_004312.2(*ARR3*):c.767+1G>A; p.? (Figure S2). Since other variants in the ARR3 gene have been linked to early onset high myopia and because the clinical findings concurred with the findings in patients described by Xiou et al., we concluded that this pathogenic variant was causative for the early onset high myopia (myopia type 26; MIM# 301010) in this family and the gene was added to the vision panel for WES. Molecular testing of the other members of this family (the older sister A, the father (C) and father's mother (E)), showed complete segregation of the *ARR3* mutation with the phenotype. As expected, the *ARR3* mutation was not present in the nonmyopic mother (D).

Since the WES vision panel had been adjusted due to the results of Family I, it included analysis of the *ARR3* gene when it was performed in family II and III. Therefore, the *ARR3* mutations in these families were recognized. In Family II, a novel pathogenic nonsense variant NM_004312.2(*ARR3*):c.214C>T, p.(Arg72*) was identified, showing segregation in two other affected females of this family (Aunt D and niece G) (Figure S2). A frameshift mutation, NM_004312.2(ARR3):c.848delG, p.(Gly283fs), was found in the index patient of family III (Figure S2). The pathogenic variant segregated in her affected sister (C).

As a next step, we determined the occurrence of *ARR3* mutation in our diagnostic vision panel WES series of 75 high myopia index patients, including the three probands described above. In this cohort, a pathogenic variant in *ARR3* had been identified in four index patients (5%). In the fourth index patient, the variant was inherited from the father without myopia. Unfortunately, further segregation analysis could not be performed but family history did not show myopia. Therefore definite causality of the variant could not be determined.

## 4 DISCUSSION

In this study we describe three Caucasian multigenerational families with early onset high myopia with an X‐linked female‐limited inheritance pattern caused by a mutation in the *ARR3* gene. The myopia phenotype in carriers of this *ARR3* variant was characterized by an early onset (<6 years), a progressive nature and a moderate to bad response to atropine treatment. In contrast to a regular X‐linked disease, high myopia was only present in female carriers. Identification of the inheritance pattern during first presentation by drawing a family pedigree will increase early recognition of this type of myopia and as a consequence will lead to diagnosing *ARR3*, improve genetic counseling and treatment initiation. Causal pathogenic variants in *ARR3* were present in 5% of our patients undergoing WES with vision panel and therefore it is a relatively common cause of high myopia.

Two previous reports described the link between *ARR3* and early onset high myopia. Xiao et al. described three families with East Asian ethnicity and early onset high myopia caused by three different mutations, namely, two missense mutations (NM_004312.2(*ARR3*):c.893C>A (p.Ala298Asp) and NM_004312.2(*ARR3*):c.239T>C (p.Leu80Pro)) and a nonsense mutation (NM_004312.2:(*ARR3*)c.298C>T (pArg100*)), which was different from our identified nonsense and frameshift mutations (Xiao et al., 2016). Széll et al. described a single Hungarian multigenerational family of Caucasian ethnicity with the same nonsense mutation as identified in our Family II (c.214C>T, p.Arg72*) (Széll et al., 2021). Both studies described a highly myopic phenotype: the carriers in the East Asian study were all ≤−6D (not further specified) and in the Hungarian study myopia ranged from −6D to −23D. When age was taken into consideration, our probands were slightly more myopic (−8D at age 4 years and even −16D at age 9 years) compared with the youngest patient in the Hungarian family (−6D, age 10 years) and the patients in the East Asian families, although no age or exact SER was specified in the latter one. Considering the phenotype in the male carriers, astigmatism as observed in our Family I was not seen in the other studies, neither in the two other families from our study nor in the Chinese or Hungarian studies. This variability in phenotype might have been caused by different environmental exposure to near work and outdoor activity, which was unfortunately not measured in any of these studies (Enthoven et al., 2019). The phenotype of all female carriers in all three studies was characterized by myopic retinal changes ranging from tessellated fundus, that is, tigroid appearance of the retina, to diffuse chorioretinal atrophy (META‐PM 0‐2(Ohno‐Matsui et al., 2015)) although one of our patients also presented with a staphyloma which was even visible on MRI. We expect that any differences in the occurrence of other complications (retinal detachment, glaucoma, and cataract) will mainly depend on the age and axial length of the patients and not on the type of the pathogenic variant per se (Tideman et al., 2016). Further functional studies should investigate the effect of the mutation on the protein expression and its function and whether this can be modulated by environmental triggers.

**Figure 4:**
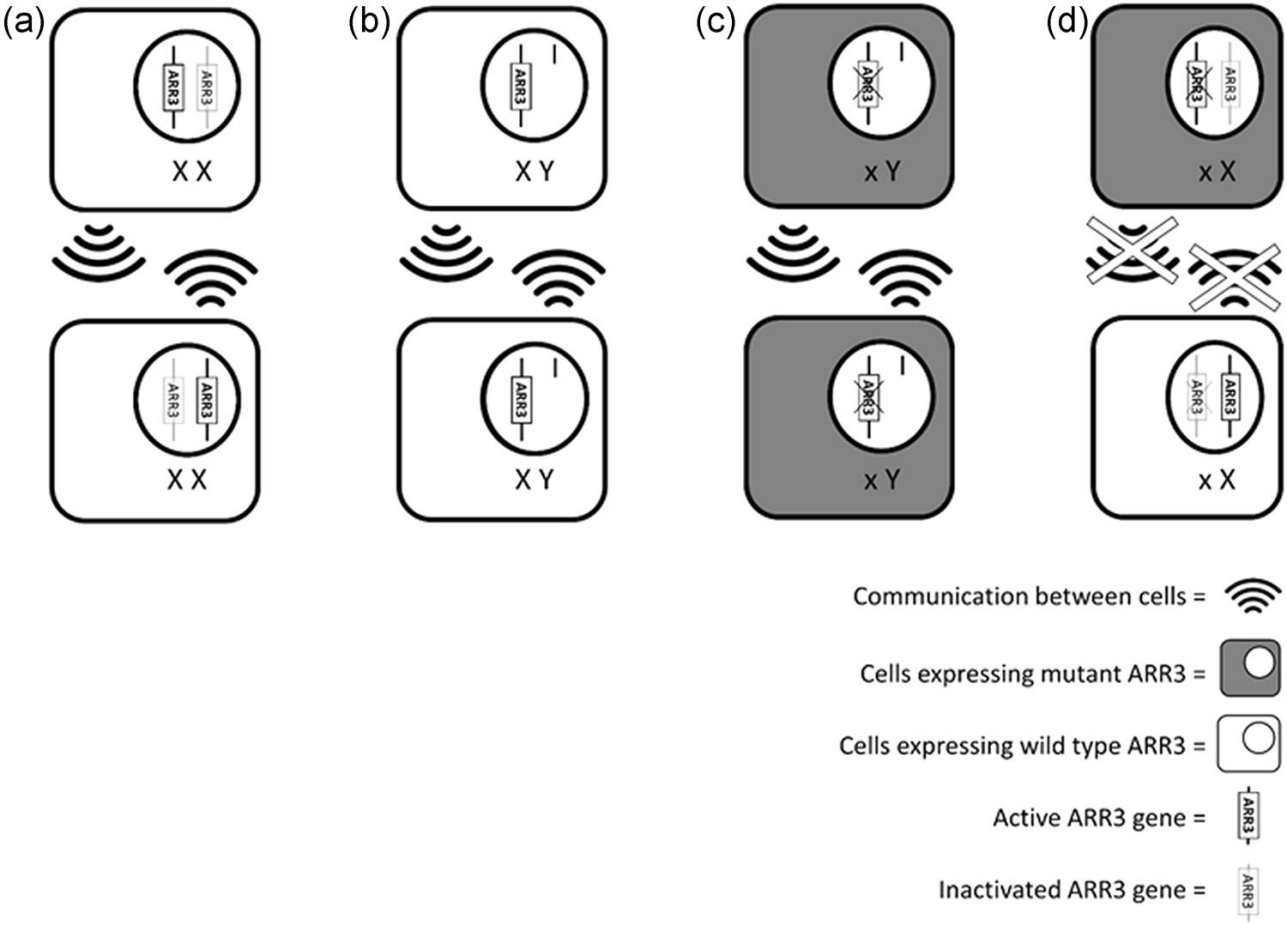
The hypothesized pathogenic mechanism of metabolic interference in X‐linked female limited ARR3 mutations. In situation A two cells in a female have inactivated different X chromosomes, the wild‐type ARR3 gene and there is normal communication between these cells. Situation B illustrates the expression of the wild‐type ARR3 in a male hemizygotes, both cells express the same ARR3 gene since males have only one X‐chromosome and communication between these cells is normal. The male cells in situation C both express the hemizygotes mutant ARR3, which allow for normal interaction between these cells despite having a mutant ARR3 gene. Lastly, situation D illustrates a female heterozygous for an ARR3 mutation: both cells express a different ARR3 gene causing mosaicism

The case of family I illustrates how a variant in the *ARR3* gene is easily missed due to its mode of inheritance. An X‐linked female limited inheritance pattern is quite exceptional and only known from craniofrontonasal syndrome caused by *EFNB1* mutations (Twigg et al., 2004; Wieland et al., 2004) (MIM# 304110) and epilepsy in females caused by *PCDH19* mutations variants (Jamal et al., 2010) (MIM# 300088). The latter syndrome is also characterized by mental retardation and a variable degree of intellectual deficit. Both are considered X‐linked of variable variants' penetrance and if inherited, they show a unique pattern of transmission. Females with heterozygous variants are affected, while hemizygous males are not or only display minor features. This singular mode of inheritance might be explained by cell interference as a pathogenic molecular mechanism leading to cellular dysfunction (Duszyc et al., 2015; Wieland et al., 2002). This suggests that there is some form of metabolic interference between the protein products of the wild‐type *ARR3* gene and that of the mutant *ARR3* gene which are active in different cells due to X‐inactivation in females (Figure 4). In males, who carry only one X‐chromosome, interference plays no role causing no dysfunction of the Arrestin‐3 protein.

We observed a moderate to bad response on atropine treatment (Family I and II) in the *ARR3* patients. This progression rate was more than earlier reported in European children (Polling et al., 2016). Known risk factors for myopia progression during atropine treatment include; young age, high degree of myopia, and Asian ethnicity (Chia et al., 2012). The effect of atropine treatment in children with Mendelian forms of myopia is scarce. Genetic predisposition as observed in these patients could be added to the known risk factors for progression warranting better treatment strategies or even different therapy options for this group of patients with Mendelian forms of myopia.

The exact function of the *ARR3* gene in the myopisation process remains unclear. The *ARR3* gene is located at chromosome Xq13.1 and includes 17 coding exons. It encodes a cone‐arrestin (Arrestin‐3) with retina‐specific and retina‐enriched expression (Craft et al., 1994; Sakuma et al., 1996, 1998). In humans, *ARR3* was first discovered to be expressed in the pineal gland but is predominantly expressed in L/M cones (Cowan et al., 2020; Craft & Deming, 2014). *ARR3* has been speculated to play a role in retina‐specific signal transduction (Maeda et al., 2000; Murakami et al., 1993) and especially in desensitization of photoactivated G protein‐coupled receptors (GPCRs) with high preference for opsins (Craft & Deming, 2014). In knockout mice with absent *ARR3*, the responses to light by photoreceptors were prolonged (Bandyopadhyay et al., 2018; Deming, Pak, Brown, et al., 2015; Xu et al., 1997; Zhang et al., 2003). In rodents, visual arrestins influence ocular development, axial length/refraction, visual acuity, and contrast sensitivity (Deming, Pak, Shin, et al., 2015; Tan et al., 2012). Our study lacked data on electrophysiological testing, although none of the patients exhibited color vision aberrations. In the Hungarian study, diffuse color discrimination defects without a specific axis, delayed visual evoked potential responses and a reduced amplitude of both multifocal electroretinogram (ERG) and pattern ERG was detected in both female carriers with high myopia and male carriers without myopia pointing toward a mechanism of central macular dysfunction but a normal functioning cone system, opposed to cone dysfunction in animal models (Deming, Pak, Brown, et al., 2015; Széll et al., 2021). Selective cone dysfunction, however, could not be tested.

In the Hungarian Study, four potential mechanisms were suggested to explain how pathogenic variants in *ARR3* could cause myopia: a hyperopic defocus, a blue light, an ocular circadian rhythm and finally a systemic circadian rhythm mechanism (Széll et al., 2021). The hyperopic defocus hypothesis was based on the predominant expression of ARR3 in L/M cones leading to differences in sensitization of opsins to red/green stimuli potentially leading to a hyperopic defocus signal, which is a known potential trigger for myopization in animal studies (Smith & Hung, 1999; Wildsoet & Wallman, 1995). Likewise, the sensitivity for blue light, which is thought to be a protective signal for myopia (Rucker et al., 2015) could be changed due to Arrestin‐3 dysfunction. This hypothesis should be tested using cone‐specific functioning tests. The ocular circadian rhythm hypothesis was based on dysfunction of ganglion cells and in particular of intrinsically photosensitive retinal ganglion cells (ipRGCs), as a result of Arrestin‐3 dysfunction, which are important for blue light detection (via the photosensitive protein melanopsin) and circadian rhythm control (Berson, 2003; Besharse & McMahon, 2016; Chakraborty et al., 2018; Do, 2019; Ecker et al., 2010; Stone et al., 2019; Zaidi et al., 2007). The last potential mechanism was based on the role of the pineal gland and circadian rhythm. As mentioned before, *ARR3* is also expressed in pinealocytes (Craft et al., 1994), which are responsible for the release of melatonin and central regulation of circadian rhythm. So in addition to ocular mechanisms, defects in *ARR3* could also provoke a systemic melatonin‐driven signal leading to myopia.

Further in depth molecular studies on Arrestin‐3 dysfunction and its role in myopisation are required for complete understanding of how a pathogenic mutation in *ARR3* can cause the myopia phenotype, limited to females.

In summary, mutations in the *ARR3* gene should be considered in patients with early onset high myopia, especially in families with an X‐linked female limited inheritance pattern. Unless a thorough family history and pedigree interpretation is performed, this rather frequent cause of early onset high myopia can easily be missed. We advise to include the *ARR3* gene to existing WES analysis panels for myopia. Although only four other families have been described before (Széll et al., 2021; Xiao et al., 2016), mutations in *ARR3* might be a relative frequent cause of nonsyndromic early onset high myopia, because an *ARR3* mutation has been found in 5% of our cohort of high myopia index patients. It can be missed in patients without taking a careful family history. Recognition of this disorder is important for many reasons. First, it gives insights into the prognosis since this *ARR3* mutation causes high degrees of myopia, increasing the risks of visual blinding complications such as glaucoma, cataracts, retinal detachment, and myopic macular degeneration which led to the loss of independence and inability to work in adult life in some of our patients (Haarman et al., 2020). Second, it might alert ophthalmologists for early initiation of treatment and monitoring of treatment response, since rapid increase of atropine dosage might be needed (Klaver et al., 2020). Furthermore, identification of this *ARR3* mutation enables genetic counseling of the patient and their relatives.

## CONFLICT OF INTERESTS

None of the authors have financial disclosures that relate to this manuscript.

## Supporting information

Supporting information.

## Data Availability

The data that support the findings of this study are available on request from the corresponding author. The data are not publicly available due to privacy or ethical restrictions. De genetic variants described in this manuscript are available on https://eur01.safelinks.protection.outlook.com/?url=https%3A%2F%2Fvkgl.molgeniscloud.org%2F%26data=04%7C01%7Ca.haarman%40erasmusmc.nl%7Cc41a4738f4c844a4eaad08d9cc345156%7C526638ba6af34b0fa532a1a511f4ac80%7C0%7C0%7C637765347341006011%7CUnknown%7CTWFpbGZsb3d8eyJWIjoiMC4wLjAwMDAiLCJQIjoiV2luMzIiLCJBTiI6Ik1haWwiLCJXVCI6Mn0%3D%7C3000%26sdata=tLfbRGvZqzlBwho2%2FGJZpInQVRIC3K1YE9DnTnFRZqU%3D%26reserved=0 using the following identification numbers: 27a93b3cab (NM_004312.2:c.214C>T; p.R72*), 85e1082b1d (NM_004312.2: c.767+1G>A p.?) and c8c465088b (NM_004312.2: c.848delG, p.G283fs).
