# Supplementary material for: Early onset X‐linked female limited high myopia in three multigenerational families caused by novel mutations in the ARR3 gene
Source: Hum Mutat. 2022 Jan 19;43(3):380–8. doi: 10.1002/humu.24327 (PMC9303208; doi:10.1002/humu.24327)
Supplement: Supplementary file 1 — Supporting information. [file HUMU-43-380-s001.pdf]

Supplementary figures

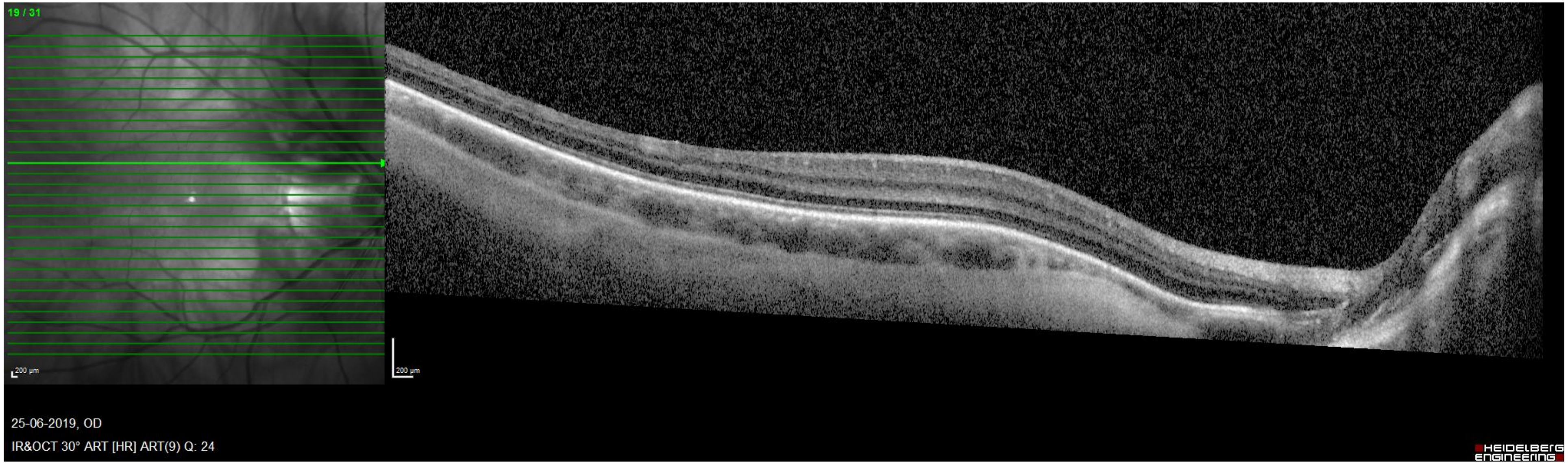


**Supplemental Figure 1:** OCT image of staphyloma of the proband of Family III.

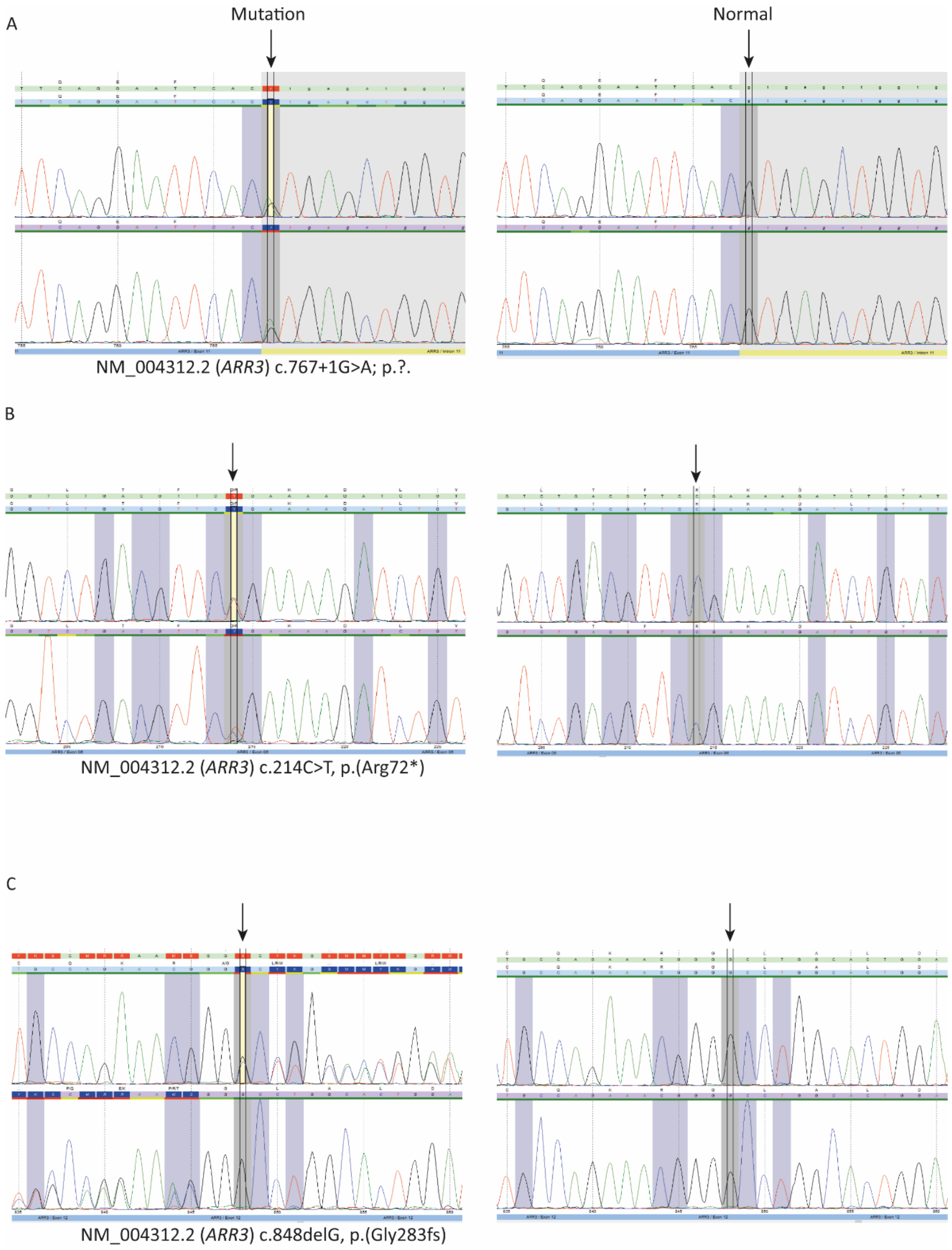


**Supplemental Figure 2:** Sanger chromatography of the three different mutations of Family I (A), II (B) and III (C)).
